# Physician–patient communication in decision-making about Caesarean sections in eight district hospitals in Bangladesh: a mixed-method study

**DOI:** 10.1186/s12978-021-01098-8

**Published:** 2021-02-09

**Authors:** Sathyanarayanan Doraiswamy, Sk Masum Billah, Farhana Karim, Md Shahjahan Siraj, Alan Buckingham, Carol Kingdon

**Affiliations:** 1 University of Bath, Bath, UK; 2 Maternal and Child Health Division, icddr,b, Dhaka, Bangladesh; 3 Faculty of Medicine and Health, Sydney School of Public Health, The University of Sydney, Camperdown, NSW Australia; 4 TEAM University, Tashkent, Uzbekistan; 5 University of Central Lancashire, Preston, UK

**Keywords:** Caesarean section, Physician–patient communication, Mixed-methods, Decision-making, Bangladesh

## Abstract

**Background:**

Caesarean sections (CS) in Bangladesh have risen eight-fold in the last 15 years. Few studies have explored why. Anecdotally, physicians suggest maternal request for CS is a reason. Women and families suggest physicians influence their decision-making. The aim of this research was to understand more about the decision-making process surrounding CS by exploring physician–patient communication leading to informed-consent for the operation.

**Methods:**

We conducted a mixed-method study using structured observations with the Option Grid Collaborative’s OPTION5 tool and interviews with physicians and women between July and December 2018. Study participants were recruited from eight district public-sector hospitals. Eligibility criteria for facilities was ≥ 80 births every month; and for physicians, was that they had performed CSs. Women aged ≥ 18 years, providing consent, and delivering at a facility were included in the observation component; primigravid women delivering by CS were selected for the in-depth interviews. Quantitative data from observations were analysed using descriptive statistics. Following transcription and translation, a preliminary coding framework was devised for the qualitative data analysis. We combined both inductive and deductive approaches in our thematic analysis.

**Results:**

In total, 306 labour situations were observed, and interviews were conducted with 16 physicians and 32 women who delivered by CS (16 emergency CS; 16 elective CS). In 92.5% of observations of physician–patient communication in the context of labour situations, the OPTION5 mean scores were low (5–25 out of 100) for presenting options, patient partnership, describing pros/cons, eliciting patient preferences and integrating patient preferences. Interviews found that non-clinical factors prime both physicians and patients in favour of CS prior to the clinical encounter in which the decision to perform a CS is documented. These interactions were both minimal in content and limited in purpose, with consent being an artefact of a process involving little communication.

**Conclusions:**

Insufficient communication between physicians and patients is one of many factors driving increasing rates of caesarean section in Bangladesh. While this single clinical encounter provides an opportunity for practice improvement, interventions are unlikley to impact rates of CS without simultaneoulsy addressing physician, patient and health system contextual factors too.

## Plain English summary

Bangladesh is witnessing a rapid increase in its caesarean section rates. In 2017/18 caesarean section rates were 33%, representing an eight-fold increase from 2004. We aimed to study factors influencing decision-making for caesarean sections in eight public sector hospitals of Bangladesh with a focus on physician–patient communication between July and December 2018.

By observing 306 labour situations and interviewing 16 physicians and 32 women who underwent emergency and elective caesarean sections, the study was able to identify that communication between the physician and the woman in the labor situation was both minimal in content and limited in purpose.

The study finds that there are factors that prime the patient and the physician in favour of caesarean section, prior to the clinical encounter. A fear of abuse and harassment by family members and general public and resulting professional disrepute; the need to preserve leisure time, including that for their families; and time for private practice to maximize income, seem to influence physicians’ attitudes towards caesarean sections. The study has identified that misinformation about childbirth is prevalent among women and their communities. Multiple providers including those who perform Ultrasonogram in rural areas are providing incorrect and inconsistent information, which leads women to have a false confidence about the safety of caesarean sections.

Health system weaknesses, particularly human resource and infrastructural challenges in providing quality emergency obstetric care, push both the physician and the patient towards caesarean sections. These identified factors, if addressed systematically, can help improve caesarean section decision making in Bangladesh.

## Background

Bangladesh is experiencing a rapid rise in the rate of caesarean section (CS). In 2004, four per cent of all births were by CS [1]. By 2018 Bangladesh’s CS rate had increased to 33% [2]. The 2018 Lancet series on CS identified Bangladesh as the country with the highest intra-institutional CS rate [3]. A CS can be life-saving for mother and child when obstetric complications arise, but the risks associated with CS, particularly when performed without a medical indication, are significantly higher in low-resource settings such as Bangladesh [4–6]. Available evidence from the global literature suggests that reasons for the increase in CS varies from country to country [7] with a complex interaction between physician, patient and health system factors [8]. There have been few studies investigating what is influencing CS rise in Bangladesh [9, 10]. These studies were only able to establish the high CS rates in the facilities studied and point to the lack of evidence-based practice underpinning CS decisions.

Healthcare delivery is profoundly affected by decisions jointly made by patients and their treating physicians. Healthcare, in general, is about decisions jointly made by patients and their treating physicians. The decision that is eventually made in the best interest of the patient is influenced by the effectiveness of communication between the patient and the physician [11]. In maternity care, WHO recommendations on antenatal care for a positive pregnancy experience advocate effective communication should be facilitated at all antenatal contacts [12]. Likewise, WHO recommendations on intrapartum care for a positive childbirth experience recommend effective communication between maternity care providers and women in labour, using simple and culturally acceptable methods [13]. The right to information, informed consent and respect for choices and preferences are critical components of respectful maternity care [12–14].

Informed consent to procedures is a key part of the shared decision-making process. The Royal College of Obstetricians and Gynaecologists (RCOG) define consent as a “process during which the professional provides accurate information concerning a procedure to a patient that allows them to reach a considered action” [15]. Similarly, the American College of Obstetricians and Gynaecologists call for effective and compassionate communication to strengthen the physician–patient relationship [16]. Despite the presence of several structured models to strengthen physician–patient communication, compliance with such models remains low [17]. Physician–patient communication has been found to be particularly weak in developing countries and in public services [18–22].

In developed countries, patients are increasingly unwilling to be passive recipients of information and are demanding information exchange with physicians, thereby potentially questioning their expert authority. In contrast, physician–patient communication and hence decision-making is still dominated by the biomedical model in developing countries. Irving et al. (2017) [23] in documenting international variations in primary care physician consultation identified that physicians in Bangladesh on average spend only 48 s with their patients, the lowest in the world identified in that review. Though not in the context of obstetric practice, this finding on low consultation time raises concerns about the physician–patient communication culture in Bangladesh. Short consultation times have been associated with poor communication with patients [24, 25]. Physician–patient communication, though, which is seen as the heart and art of medicine that shapes decision-making in CS, remains largely unexplored in the Bangladesh context.

We aimed to (a) examine the communication between physicians and patients in the period leading up to obtaining consent for emergency CS—by direct observation of deliberations that happen during labour between physicians and pregnant women (b) to study factors influencing physicians and patients in the consenting process of caesarean sections—through in-depth interviews with physicians and patients who had undergone emergency and elective CS. We chose to restrict our study to observing physician–patient communication in the lead up to CS decision making as doctors take the final decision for CS in Bangladesh [26, 27].

## Methods

This study is reported according to the ‘Good Reporting of a Mixed Methods Study’ (GRAMMS) guidance [28] (Additional file 1: Annex 1).

### Study design

We conducted a convergent parallel design mixed-method study [29] using structured observations, and in-depth qualitative interviews with physicians and women, between July and December 2018. Our intent in utilizing this design was to unite the strengths of the two methods and to compensate for weaknesses. Both methods were given equal importance in the study. The qualitative component followed the quantitative component of the study. This study was part of a larger study looking at multiple factors influencing decision-making around CS. Details of the larger study are provided in Additional file 2: Annex 1.

### Study setting and participants

All public-sector hospitals in Bangladesh, conducting at least 80 births every month, were eligible for inclusion. Of the 64 district hospitals (DH) nationally, 45 met this criterion. Microsoft Excel was used to randomly select one district hospital from each of the eight administrative divisions. This process ensured geographic spread across the final eight included hospitals. All hospitals studied were in the rural districts and on average conducted 1000 deliveries a year, sharing similarities in their catchment population, bed capacity and human resource structure. Further details about the sites can be found in Additional file 2: Annex 2.

All physicians performing CS in the selected hospitals were invited to participate in the interview component. All pregnant women attending one of the included hospitals during the study period, aged 18 years or older, and providing consent, were eligible for inclusion in the observation component; primigravid women delivering by CS were eligible for the in-depth interview component. A purposive sampling frame of 32 women was used to ensure equal numbers of women (16 each) who had an emergency CS (in labour) and women who had an elective CS (pre-labour). We included primigravid women only for the qualitative component, as a previous study in Bangladesh had found near universality in choice of elective CS amongst multigravid women. [9]. We aimed to recruit a minimum sample of 296 women but were able to reach a sample of 306 in the planned study period. This sample estimate was based on the expected number of births in the facilities for two weeks (the logistically feasible period that could be spent in each facility).

### Data collection

Labour observations happened in the labour ward and its environs where there was an interaction between the physician and the pregnant woman (and often their companions). The communication between client and the service provider, and the decision-making process for the mode of delivery was recorded using the OPTION 5 tool. This is a validated tool which measures the extent clinicians involve patients in decision making and has been used in similar studies [30]. The tool is framed around the ‘three-talk’ model of shared decision-making [31]. This model outlines the types of talk in the shared decision-making process, namely ‘team talk’, ‘option talk’ and ‘decision talk’. Five domains in decision making are measured, namely presenting option, patient partnership, describing pros/cons (advantages and disadvantages), eliciting and integrating patient preference. The tool and its scoring have been included in Table 1.

**Table 1:** The observer OPTION 5 Measure- Score Sheet

*Item 1*: For the health issue being discussed, the clinician draws attention to or confirms that alternate treatment or management options exist or that the need for a decision exists. If the patient rather than the clinician draws attention to the availability of options, the clinician responds by agreeing that the option need deliberation 0 = No effort 1 = Minimal effort 2 = Moderate effort 3 = Skilled effort 4 = Exemplary effort
*Item 2*: The clinician reassures the patient or re-affirms that the clinician will support the patient to become informed or deliberate about the options. If the patient states that they have sought or obtained information prior to the encounter, the clinician supports such deliberation process 0 = No effort 1 = Minimal effort 2 = Moderate effort 3 = Skilled effort 4 = Exemplary effort
*Item 3*: The clinician gives information or checks understanding about the options that are considered reasonable (this can include taking no action), to support the patient in comparing alternatives. If the patient requests clarification, the clinician supports the process 0 = No effort 1 = Minimal effort 2 = Moderate effort 3 = Skilled effort 4 = Exemplary effort
*Item 4*: The clinician makes an effort to elicit the patient’s preferences in response to options that have been described. If the patient declares their preference(s), the clinician is supportive 0 = No effort 1 = Minimal effort 2 = Moderate effort 3 = Skilled effort 4 = Exemplary effort
*Item 5*: The clinician makes an effort to integrate the patient’s elicited preferences as decisions are made. If the patient indicates how best to integrate their preferences as decisions are made, the clinician makes an effort to do so 0 = No effort 1 = Minimal effort 2 = Moderate effort 3 = Skilled effort 4 = Exemplary effort

Score	Description
0 = No effort	Zero effort observed
1 = Minimal effort	Effort to communicate could be implied or interrupted
2 = Moderate effort	Basic phrases or sentences used
3 = Skilled effort	Substantive phrases or sentences used
4 = Exemplary effort	Clear, accurate communication methods used

Three experienced female physicians who had worked in a similar study before were deployed to collect data for this component of the study. It was deemed culturally more acceptable to have women directly observe deliveries in the context of Bangladesh. For the qualitative interviews, separate schedules were developed for physicians and women who underwent CS. Interviews were conducted by six trained, experienced psychologists and anthropologists in total. Teams of two members went to each of the sites and had one supervisor to assist when needed. There were no prior relationships between the interviewers and the participants. Physicians and women were interviewed in the hospitals. Interviews varied in duration from 45 to 90 min. Interviews were conducted before women left the hospital (most commonly around day 3). Interviews were conducted during non-visiting hours to provide maximum privacy to the mother. Interviewers were trained to stop the interview if they witnessed any discomfort on the part of the mother. The mothers who had an adverse birth outcome were not interviewed. All interviews were recorded in Bangla and later transcribed in English. The interview guide is included as Additional file 3—Annex 1.

### Data analysis

In accordance with the convergent parallel design, data were analysed separately prior to an integrated interpretation of the results. Range and consistency checks were conducted on the quantitative data, and cleaned data were transferred into Stata® v13.0 [32] by FK and SS. Analysis principally used descriptive statistics. A summary score of communication between service provider and client was calculated by adding scores of all five domains of OPTION5 tool for each encounter. Mean difference of total score and that of each domain between normal vaginal delivery (NVD) and CS clients was calculated. Independent sample t-test was used to explore if the mean score differences between the two groups were statistically significant at a level of p < 0.05. Audio recorded in-depth interviews of both physicians and patients were transcribed verbatim into Bangla by trained transcribers, immediately after the interviews. After completion of transcription and translation into English, the analysis was done on line-by-line content using thematic analysis strategies [33]. Both deductive and inductive coding techniques were combined. In order to increase reliability, a quarter of the interviews were randomly selected and were independently coded by another trained qualitative researcher. Integration of data was led by SD, in conjunction with SMB, AB and CK.

## Results

**Fig. 1:**
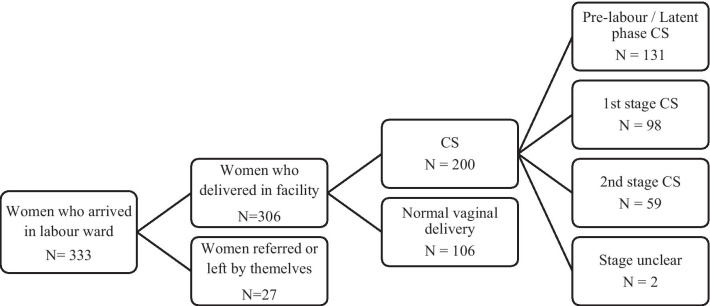
Flow diagram of the participants in the observation study component

333 women arrived in the labour ward of the selected facilities during the period of the study. Subsequently, 27 participants either chose to leave the facilities themselves for care elsewhere or were referred by the treating physician to higher level of care. This left 306 who were observed until the point of normal delivery or consent for CS. The number of deliveries observed in each of the hospitals ranged from 23 in the least-busy hospital to 61 in the most-busy hospital (median – 40 deliveries). Two hundred (65%) of these women delivered by caesarean section and 106 women had a vaginal birth (See Fig. 1). Out of the 200 CS, 131 of them were done pre-labour or during the latent first stage of labour; 8 deliveries were CS at 1st stage of labour and 59 were CS at 2nd stage.

Table 2 shows the characteristics of the participants whose labour situations were observed. Most of the women were in the age group of 19–24 and had primary education or less. Out of the 306 women observed, 111 (36.3%) women were primigravid. 44.4% of the women reported to have a family income of less than USD 150 per month (1 USD = 85 Bangladesh Taka). Other studies have established that public facilities were usually visited by populations in the poorest quintiles [34].

**Table 2:** Characteristics of women, whose labour situations were observed

Characteristic	Number (%)
Age	306(100)
18–19	32 (10.5)
19–24	116 (37.9)
25–29	100 (32.7)
30–34	52 (17.0)
> 35	6 (2)
Education	
No education	39 (12.6)
Grades 1–6	134 (43.8)
Grades 7–12	76 (24.9)
College	57 (18.6)
Family income (USD) per month	
< 150	136 (44.4)
150–250	107 (35)
> 250	63 (20.6)

The OPTION 5 tool scored the five domains of shared decision making between the service provider and the pregnant women in the labor setting. Each observation is given a score of 0–20 and then multiplied by five to give a score out of 100 as outlined in Table 1. A score of 100 denotes exemplary effort in the shared decision-making process while a score of zero implies no effort at all.

The majority, 92.5% of the encounters in this study, scored less than 25% of the maximum score. Just over ten per cent (12.1%) scored zero. The overall mean score for OPTION 5 was 14.9 out of 100. The OPTION5 item wise mean scores for presenting options, patient partnership, describing pros/cons, eliciting patient preferences and integrating patient preferences are shown in Table 3. In line with the overall score, individual scores across all the five domains were very low. Since the observation data includes both CS and normal deliveries, further analysis was done to see if there was any statistically significant difference in the patterns of shared decision-making in CS and normal vaginal deliveries (NVDs). We used the t test for independent means to test for significance. When the mean difference of the overall scores of the two groups were compared there was no statistically significant difference (p-value < 0.05). Among the individual domains, we observed a statistically significant difference only in the pros/cons domain.

**Table 3:** Mean OPTION 5 scores

Item	All encounters (N = 306)	NVD (n = 106)	CS (n = 200)	Mean difference (95% confidence interval)
Item 1 (presenting options)	5.03 (± 2.38)	5.24 (± 2.88)	4.93(± 3.03)	0.31(-1.01,0.39)
Item 2 (patient partnership)	4.02 (± 2.15)	3.96(± 2.82)	4.05(± 2.62)	0.09(-0.55, 0.72)
Item 3 (describing pros/cons)	2.48 (± 2.20)	1.84(± 2.70)	2.83(± 2.73)	0.99(0.34, 1.63)*
Item 4 (eliciting patient preferences)	1.90 (± 2.34)	1.32(± 2.42)	2.20(± 3.12)	0.88(0.19, 1.56)
Item 5 (integrating patient preferences)	1.49 (± 2.0)	1.13(± 2.32)	1.68(± 2.57)	0.55(-0.04, 1.13)
Total score (out of 100)	14.92 (± 10.5)	13.49(± 10.15)	15.69(± 10.64)	2.20(-0.29, 4.66)

*p value < 0.05

### Contextual factors influencing how physicians communicate during decision-making for caesarean section

Sixteen interviews were conducted with physicians. At selected study sites all the physicians performing CSs were female. Consequently, all the physician interviewees were female. The ages of participants ranged from 30 to 47 years (mean 39 years), with years of obstetric experience ranging from 4 to 28 years (mean 11 years). All participants had a degree or a diploma in obstetrics. Six participants had one child and ten had two children. Fourteen out of sixteen physicians, had their children delivered by CS. All the included physicians provided only obstetrics and gynecology services in their hospitals. The ranges of services included obstetrics and gynecology outpatient services including antenatal and post-natal care; normal vaginal deliveries and CS; gynecological surgeries and post-operative follow-up. As junior and senior consultants, the interviewed physicians were also involved in training nurses and midwives in the hospitals and also had administrative functions involving their wards and as assigned by the superintendent of the hospital. As permitted in Bangladesh, all 16 interviewed doctors were involved in private practice which included both general practice and specialized services for obstetrics and gynecology (predominantly the latter though).

Table 4 provides a summary of the, categories, emergent and organizing themes from which the principal finding *the importance of wider contextual factors on decision-making* emerged. Additional file 3: Annex 2a lists the codes and connects them with the categories, themes and contexts for each of the participant category.

**Table 4:** Categories, themes and contexts from qualitative interviews

Physicians	Categories	Personal and professional workload balance; Physician experience and perceptions; External influence; Fear and Risk Aversion; Communication as a way of sharing information; Human resource challenges
	Themes	Work-life balance; Personal preferences; External influence; Risk Aversion; Communication skills; Health system
	Contexts	From within; From without; System and skills
Women who underwent Emergency CS	Categories	Local pressure; Health workers attitude; Confidence in indications; Negative information exchange; Interpretation skills of the woman; Do what you can; Emotional drain
	Themes	Yielding to local pressure; Lack of respect; Speaking the same language on indications; Negative language; Technical language; Prayers take over; Decision under pressure/ Quick end
	Contexts	Guilt; Powerlessness; Knowledge; Language; Fatalism
Women who underwent Elective CS	Categories	Faith and resigned to a destiny; USG and its universality for determining indications; Sources of information One-way (limited) communication; Consenting without understanding; An added benefit of combining sterilization; Complications don’t matter; Lack of privacy fuelling fear
	Themes	Faith; USG and its universality; Confidence in safety Physicians know best; Consent, a formality; Collateral benefits; Baby is the future; Privacy over pain
	Contexts	Safety of C-sections; Physicians in control; Value for money; Sacrificial attitude; Fear of pain—not a major concern

The first organizing theme *from within* encapsulates factors that were intrinsic to the physician; namely, *work-life balance* and *personal preferences.* Physicians reported being overworked. They were balancing wide-ranging public-sector roles with private practice, which meant long hours, and an ongoing struggle to find time for their own families. They identified these competing demands as preventing them from spending time communicating with pregnant women and their relatives. *“I always used to work with normal deliveries… but here in this centre, I have to perform C-sections, ward rounds, and even some office work as well. It is not possible for one person to do everything, so we have to make a balance… a physician cannot attend everywhere.”*—Physician 10; *“I have to see sixty patients daily. If I have to counsel attendants of every one of them, then I won’t have time for doing operations.” Physician 7 “My child is very young, so I can’t afford much time.”*—Physician 1.

As alluded to in the quote from Physician 10, participants acknowledged that labour and vaginal deliveries take time. Time that they do not have. Participants were open about the demands on them as professionals, and as women. Working at night engendered specific concerns about personal security, lack of transport and childcare. *Personal preferences* informed by personal and professional experiences were also reported as important influencing factors, overriding international and national guidelines, which they perceived as irrelevant to their local situation. “*Sometimes we couldn’t follow the protocol exactly. We do it from our experience.”-* Physician 4.

Some physicians discussed feelings of uncertainty surrounding indications for CS and inconsistencies in practice. They were not aware of their own or their institutional CS rate, but as previously reported 14 of the 16 physicians had CSs themselves. While these participants were insistent that their CSs were for valid medical indications, there were contradictions in their accounts which suggest it was also their preference. *“It was my fault. I was a high-risk mother. I had a bad obstetric history. I had two abortion experiences. So, we didn’t want to take any risk. Though the next issue came within 13 months after the first delivery, I have to go for C-section.”*—Physician 4.

The second organizing theme *from without* encapsulates factors that were extrinsic to the physician which they reported exerted an *external influence* on how they communicated with patients in the context of CS decision-making. These external influences included co-workers, family members of patients, local politicians and journalists/mass-media professionals. The physicians felt that they faced a lot of pressure from the mentioned actors to perform CS in patients known or related to them. While the pressure was intense from all of them, the type of pressure varied. While co-workers exerted social pressure asking for a favour in the form of a CS for their friends and relatives, family members of patients resorted to verbal and physical threats as a form of exerting pressure. Among all the actors, the physicians repeatedly mentioned local politicians and journalists as those capable of bringing professional disrepute to them by foisting false stories about them resulting in professional disrepute and/ or job transfers if physicians do not comply with their request for CS. *“Of course, the patient of a journalist is like the political person. They force me to do caesarean at 3 a.m. They are very dangerous. Nowadays, there are so many journalists. Easily they become a journalist. It’s become a phobia to us.”—*Physician 11.

The third organizing theme *system and skills* encapsulates three emergent factors; *risk aversion*, *communication skills* and *health system factors*. Risk aversion is viewed as defensive obstetrics, specifically thinking of the worst possible outcome in each instance and protecting one’s self from blame and repercussions. This is seen as a critical factor behind the CS decision-making process. The risk and fear come less from litigation [35], unlike in the Western world, and more from physical threats and professional disrepute. The physicians seem to acknowledge their limitations in communication skills. They experience minimal training during their medical education and learning from teachers during ward rounds, but they wanted more formal communication training. The physicians cited many challenges in the physical infrastructure, manpower, availability of supplies and support personnel. These constraints also had a bearing on their CS decision-making. *“We do not have enough anaesthetists. So, it has become a kind of official order that sirs (anaesthetists) are to inject anaesthesia only in the morning, not in the evening or at night. So, we do not have an operation theatre in the evening or at night.”*—Physician 1*; Counselling (i.e. giving advice) is a part of our academic study. That’s what we call communication part. Communication with patients is very important and if there is any training in this regard, then it is easy to handle the patients. –* Physician 7.

In summary, the physicians were under pressure from within, without and the systems they operate within. All these factors have rendered the physicians ‘risk averse’ and had a major impact on their communication with women and their families.

### Contextual factors influencing how women perceive their role in decision-making about emergency CS

The majority of women interviewed (7/16) were in the age group of 19–24 (mean age 22.9 years). The majority of women (7/16) had 1–6 years of schooling only. All women were primiparous. Additional file 3: Annex 2b shows the codes and themes from which the principal finding of *the broader influential factors on patients* had emerged.

The first organizing theme of *guilt* comprises the reasons behind the women *yielding to local pressure* exerted by the healthcare environment they found themselves in. One explanation for such feelings of guilt was in the current, as expressed by this woman; “W*e were bound to take the decision to have a CS. We wanted to have a normal delivery at home. We tried by the traditional birth attendant at home and it failed; we were afraid”—*Emergency CS patient 15*.* Feelings of guilt were also future orientated, faced with the prospect of not consenting to CS and their baby subsequently dying.

The second organizing theme of *powerlessness* was driven by a *lack of trust* between the patient and the physician. In most instances, the women had visited multiple health facilities and had seen many health care providers before they arrived in the health facility where the CS happened. A sense of mistrust was perpetuated by a lack of respect, empathy and care from the staff in the short time the women were there. The women were rendered powerless to express their preference either in fear to speak up or believed that it was pointless communicating their wishes to the staff. *“How could we (discuss our preferred mode of delivery)? Is it possible to tell physician everything? Why didn’t we tell? We were afraid; it’s not possible to say so many things” –* Emergency CS patient 10.

The third theme that emerged was women’s *knowledge* about indications for CS. Women and their families seemed pre-sensitized to some common reasons’ physicians performed CS and appeared agreeable to CS when they heard these indications from their physician. Variations in blood pressure, no fluid in the baby sac (oligohydramnios), big baby, baby in the foot or bottom presentation (breech) and short stature of mother are some of the indications that appeared frequently in discussions. Women obtained this information prior to coming to the hospital from various sources including the internet, those who had a past CS, from their radiologists who did ultra-sonograms at various stages of their pregnancy, traditional healers and others community members. While it is a well- established fact that breech presentation is common in the early stage of pregnancy and the baby’s position can change later, in the mind of the mother, this remained deep-rooted and set an expectation on the need for CS. *“Then I did ultra-sonogram on 7th month to know baby’s condition. After going there, they reported baby’s position was breech then.”*—Emergency CS patient 6.

At the same time as women were familiar with some of the common justifications for CS, the fourth theme of *language* comprised how the *negative and technical language* used by health care providers caused distress to women and their families. The language in the health care settings was either too intimidating or too technical for the women, who were young and often came from poor and low-literacy backgrounds. Agreement to the CS procedure sometimes occurred due to fright or technical intimidation. As evident in this quote, some technical messages were not given directly to the woman in labour, but rather to their relatives, or in discussion between physicians and other healthcare providers, which the women sometimes overheard; *“She told my sister, asking me to go out of the room, that it would be difficult to save my baby and me. She frightened my sister by saying this.”*—Emergency CS patient 13.

This, in turn, led to the final theme of *decision under pressure*, which included a sense of *fatalism* due to either from lack of financial resources to explore alternates or to simply get relief and bring a *quick end* to the immense pressure generated by the situation. The quote from the woman below encapsulates both the pre-existing knowledge women brought to bear and the pressure they were under in the moment. *“I was afraid of it. I always prayed to Almighty to have a normal delivery at home instead of having a hospital delivery. But Allah has brought me here to have this baby*”—Emergency CS patient 3; “*That physician suggested to do C-section and told us to let them know our decision within 5 min. I prayed to the Almighty for whatever was better to happen. If C-section is required, why delay? We proceeded.”*—Emergency CS patient 10

### Contextual factors influencing how women perceive their role in decision-making about elective CS

Half the (16) women interviewed were in the age group of 19–24 and had completed their primary education. All women were primiparous. The common indications for CS in the words of the women were ‘date over’/‘not getting pain’; ‘previous stillbirth’; ‘wrong position of baby’; ‘no water inside’. ‘Small baby’; ‘my request to combine CS and tubectomy’; ‘rising heart rate of baby’ were given as the reason by one woman each only. Detailed codes, categories, themes and contexts obtained from the interviews are provided as Additional file 3: Annex 2c.

The first organizing theme among women who underwent elective CS surrounded the perceived *safety of CS*. This theme encompassed emergent themes of *confidence in safety of CS, ultrasonogram (USG) and its universality and faith.* It was observed that some of the women had derived their confidence in the safety of CS by witnessing their friends and relatives have a CS and recover fully. As one woman reported; “*My friends also had a CS operation, it was safe and for this reason, I had the desire of doing mine”-* Elective CS patient 5. Some women reported that the only risk they knew of as associated with CS were the challenges in doing daily chores for some time. The women’s confidence that CS was safe was linked to their faith and sacrificial attitude towards motherhood. “*Almighty knows everything that would save (my) baby*”—Elective CS patient 6.

Alongside women’s religious beliefs was a belief in technology. Ultrasound scans (USG) in particular. USG was found to play a crucial part in convincing women of the need for CS. There was near universality in the use of USG among the interviewed women. Some women had up to four USGs during the course of their pregnancy. Breech presentation during the early stages of pregnancy, low amniotic fluid index, big baby and other similar indications seem to be planted in the minds of the women as ‘high risk’ for their babies, which was amplified as they approached term. “*In the one and half month of my pregnancy once I did ultrasonography in private (private clinic). Again, I did it in the third month and made a card, again, in the eighth month and in the ninth month after getting admitted. Total four times—baby was upside down and I knew I will need CS.”-* Elective CS patient 9.

Above all, the confidence in safety was derived from various forms of faith, mostly religious but also some traditional beliefs, making it a recurrent theme. This gave them confidence in the CS decision as they had resigned themselves to the fact that what was happening was due to divine will or as a counter to evil forces as indicated by some traditional healers. “*When I was pregnant, then the Kobiraj (traditional healer) warned me that some evil spirit wanted to harm me any time in the dusk. He also told me that the evil spirit passed over the roof of my house. He also could foretell that once I had gone to my relative’s house and during my pee, I did not cover my head. And since then, that the evil spirit had been after me to harm my body.”—*Elective CS patient 3.

The second theme that emerged from this group was placing the *physicians in control* of final decision-making. The act of *giving consent was seen more as a formality* by most women as they were not even aware of the purpose of signing the consent form. As one woman said, “*I don’t know for which reason they took signature. I just signed*.”—Elective CS patient 4.

The women believed that *physicians know best* and are too powerful for them to discuss preferences. Only one of the women’s comments led to the theme of *collateral benefits*. She spoke of the financial advantages of being able to combine CS and tubal ligation in one care episode. This decision seemed to have been made early in their pregnancy. *Lack of adequate privacy* as an important *health system constraint* was the final theme that emerged, as women were forced to consent to CS seeing other women experience labour pain in the facilities. *“A girl became very sick at the time of having a normal delivery. Everyone got afraid after seeing it. I will not be able to tolerate it. Then the physician examined me and was having an angry mood. She said, “We are trying to have a normal delivery. Hmmm, if you all have so much problem and want to have caesarean delivery, then we will do it*”—Elective CS patient 5. This is demonstrative of a health system not able to provide respectful maternity care to the pregnant women and hence fail to gain her trust (a theme that also emerged from the interviews who underwent emergency CS).

Looking closely at the themes that emerge from the interviews with women who underwent emergency and elective CS, there is a dominance of the underlying anxiety among the patients making them consent for C-sections. In addition, the interviews with both the physicians and the patients demonstrate the limitations of the health system, leading to further constraints in their communication.

### Integration of patient, physicians and health system factors

**Fig. 2:**
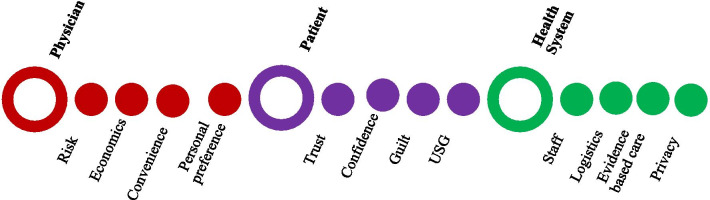
Factors influencing CS decision-making—*The priming factors*

**Fig. 3:**
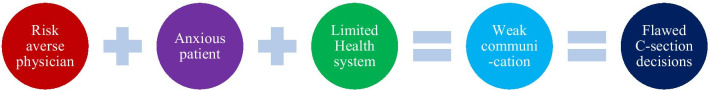
Factors directly impairing communication

We have adapted and summarized the themes from the physician and patient interviews in Fig. 2 and call them as priming factors. Collectively, these ‘priming factors’ have dominated the context in which the decisions on the observed CS were made. Figure 3 provides a summary schematic representation of this flawed decision-making process for CS. This suggests the risk perception of the physician of the harm of not performing a CS, the anxiety and lack of trust of the patient in the willingness of physicians to perform normal vaginal deliveries, in the context of a constrained health system, are contributing to flawed CS decision-making in public sector hospitals of Bangladesh and hence driving the high CS rates.

## Discussion

This study sought to understand more about the decision-making process surrounding CS by exploring physician–patient communication leading to informed-consent for the operation. The OPTION 5 instrument has been recommended and widely used in clinical settings where there is scope for shared decision-making [31]. The OPTION 5 tool is specifically used to assess the extent to which health care providers involve patients in decision-making has been used in other clinical settings [30]. To our knowledge this is the first time the OPTION 5 tool has been used in obstetric observations. We found the tool easy to use in most encounters and interpret as was identified by Ijaz et.al (2018) in a study in an emergency department [36]. While the tool has been proven to provide a better understanding of the extent of shared decision than self-reported measures, there would be difficulties when there are multiple and staged decisions [37].

We found that the physician–patient interactions were both minimal in content and limited in purpose, with consent being an artefact of a process involving little communication. The consent form is the key document around which the decision of informed consent by the patient after a discussion with a physician is supposed to pivot. The study finds, through the very low OPTION 5 scores in all domains, that the consenting process neither involves provision of accurate information nor is there a considered decision taken on the part of the patient. The statistically significant difference we found in the discussing pros and cons domain is not surprising given the effort that physicians seem to putting in convincing patients to undergo CS. This effort has no value without offering options and elicting patient preferences. The evidence supports existing research that informed consent is used as a means for preserving physician’s reputation and not primarily as a tool for shared decision making [38].

Though evidence-based and in line with universal maternal health rights, the study finds a low-level of compliance in rapport building communication, a key component of respectful maternity care[13]. This should be considered an opportunity lost to gain the trust of the women in the health system and by the health care providers not just in the context of C-section decision making but in the provision of intrapartum care. According to WHO 2015 estimates, Bangladesh currently spends US $26.60 per person on health per year. Close to two-thirds (64%) of these funds come through out-of-pocket payments [39]. The public health facilities have been hit by chronic shortage of staff and essential commodities as identified by the Bangladesh health facility survey 2017 which compromise on the provision of optimum quality of care [40]. Lack of quality of care in facilities further compromise trust. Trust in the health system is seen as an important requisite for gaining the confidence of women to participate in shared decision-making [41]. Islam and Jhora (2012) [42] identify lack of trust to be a widespread limitation in Bangladesh, in particular when it comes to caring for the poor. In their review of the physician–patient relationship in Bangladesh, they emphasize the physician–patient relationship as being the foundation of contemporary medical ethics and underscore its criticality in providing quality health care services. They identify that maintaining a professional relationship, upholding the dignity of patients and prioritizing their privacy are generally deficient in Bangladesh.

The study finds that there are multiple non-clinical factors that prime the patient and the physician in favour of CS even before the clinical encounter and there is very little evidence of any remodelling of these primed decisions during the encounter to change course. Instead the clinical encounter and the poor communication that was found to happen during it, risks setting up a vicious cycle, exaggerating the priming into a dominant form of practice with the consequence of further increasing C-section rates in Bangladesh. The role of non-clinical factors in influencing C-section decision making has also been studied in Egypt, Scandinavian and Ango-American country contexts [43–46]. These existing studies have looked at the non-clinical factors solely from the lens of the patient (e.g. avoidance of pain, convenience, greater safety of the baby), from that of the physician (incentives, lack of supervision, lack of familiarity with guidelines) or the health system. Our study, while confirming some of these already known factors in the Bangladesh context, adds new detail bringing in both the physician and patient dimensions together and exploring the role of health system in influencing both the physician and the patient.

An unexpected finding is the universality of ultrasonogram (USG) investigation. A recent study in one of the rural districts similar to the districts in our study found an USG coverage of 45% in pregnant women (at least one scan during the course of the pregnancy) [47]. The Obstetrics and Gynecology society of Bangladesh recommends one USG during weeks 24–28 to detect fetal anomalies. Two other scans are recommended (one before 16 weeks and the other 36–38) weeks only where available. USG in weeks 32 is advised if any complication is suspected [48]. All women interviewed in our study had at least one USG done during their pregnancy, and some of them had up to four USGs during the course of their pregnancy. USG at the early stages can identify presentations such a breech (though the purpose of USG in those stages is for determining viability of fetus and to detect fetal anomalies only), which is likely to correct itself during the course of the pregnancy. A sense of fear seems to be instilled in the minds of women based on such findings in the USG and women tend to carry this as a high risk all the way up to delivery, and it seems to influence their eventual decision-making.

The role of power differentials and stakeholder commitment has been well documented in the existing literature [49]. The results from the study also resonate with existing literature [50] on the complexity of communication between the physician and the patient (in most cases, the pregnant woman and her family) in the context of consent for CS. In this study, it has been possible to delineate these factors and group them under physician, health system and patient factors. The risk aversion of doctors and a practice of defensive obstetrics has been documented in other studies [51]. The growing risk of violence for physicians at the hands of patients and their relatives is a growing phenomenon worldwide [51, 52] and has been well documented in China [53, 54]. The study agrees with many women and communities, health professional and health organization related factors identified by Betran et.al (2018) in their contribution to the Lancet series on CS [8]. For women, these factors include considering CS as safe procedure for themselves and their babies and the fear of suboptimal quality of care and fear of disrespect and abuse in the health facilities. The concept of CS being ‘safe’ is also shared by the physicians in our study. This sentiment seems to prevail not just for patients but also for themselves. Existing studies of professional and personal preferences have been inconsistent in reporting female doctors’ greater preference for CS [55–57]. Several studies report female gender is associated with a lower likelihood of accepting the woman’s request for a CS, especially when female doctors themselves have had children [58, 59]. The fact that majority of the interviewed physicians (14/16) in this study actually had a CS themselves is demonstrative of the confidence in obstetric interventions amongst physicians in this setting. A similarly high prevalence of CS among female doctors was found in a study in Iran [57].

Though religious considerations have generally been found to be barriers for women accepting C-sections in other studies [60], religious and spiritual beliefs have also been shown to increase self-efficacy in some studies [61]. This self-efficacy drawn from religious faith has been found to influence women to agree to C-sections. In our study, we did not find fear of labour pain to be one of the reasons for women agreeing to CS. Also novel in our study is the identification of ‘guilt’ as a consequence to attempting delivery at home as one of the reasons for women agreeing to CS. It is to note that Bangladesh experiences a high home delivery rate of around 50% [2]. Lack of adherence to standard guidelines and the role of external pressure on physicians have been identified as the reasons for CS in similar studies in Bangladesh [10]. This would be an important focus for future research.

The 306 women who were observed in the labour ward included 111 (36.3%) nulliparous women and 205 (63.7%) multiparous women. This proportion is similar to what Begum et al. [22] found in their population-based study in Matlab, Bangladesh (41.3% nulliparous vs 58.6% multiparous). The observed intra-institutional CS rate in our study was 65% (the proportion of live births by CS within health institutions). This CS rate is similar to the figure cited for Bangladesh in the global analysis of CS rates by Boerma et al. [3].

## Strengths and limitations

The study draws its strengths from its mixed-methods research design, through triangulation and complementariness of the two forms of data. The purposive sampling used for the quantitative component of the study is a limitation, as the available logistics only permitted us to spend two weeks in each facility. This measure though permitted us to reach hospitals across the entire country giving us a wide geographic spread. The eight target hospitals are representative of all district hospitals in the country and represent all administrative divisions of the country. Though we tried to obtain the monthly family income from the women included in our study, this was not sufficient to determine wealth quintiles that the women belong to. However, the data obtained was sufficient to find that most of the interviewed women belonged to the poorer section of the society. The study did its best to distinguish emergency and elective CS to see if there are any differences in decision-making, although it was recognized that in practice this distinction was difficult to make, and a few cases might have been wrongly categorized. It is a limitation of this study that records of medical indications for CS were not triangulated with the interview data. The study only focused on primary CS. The study only explores the perspectives of women who had undergone CS, and how decisions were fashioned, and it did not deal with the preferred modes of delivery or the views of women experiencing labour and vaginal birth. It may be considered a limitation that women were interviewed before discharge from hospital, following major surgery. At the same time, it is a strength of this research that it sought to capture women’s views and experiences to triangulate with the professional interviews and structured observations.

All the clinicians interviewed were women and mothers, most of whom (14/16) delivered by caesarean section. This may be considered a limitation. Furthermore, the study does not obtain perspectives of nurses, who seem to play an important role in the communication that happens in labor situations. Midwives are a new profession in Bangladesh and can be expected to play a dominant role in the future. Quality of care, women’s experiences of vaginal birth and the role of midwives are important areas for future research.

## Conclusion

The study has established that the physicians and patients arrive at the clinical counter with their minds already primed for CS. CS has established itself as a dominant form of practice in Bangladesh. The consent form has become an artefact of a prior process, and the data in this study demonstrates that the decision comes about through factors outside of the formal consent process, though the clinical encounter and the physician–patient communication provides an opportunity to reduce the priming effects. Lack of meaningful communication is a lost opportunity for reducing these priming effects. Greater investment is needed to sensitize women and communities about the risks of CS that are not medically indicated and the benefits of adhering to expert medical advice only. Efforts should be made to dispel myths and misconceptions around services provided in public sector facilities, to empower the communities to know their rights and to be confident in discussing options with physicians. The gaps in the health system with respect to human resources, physical infrastructure, equipment, and supplies, ensuring physical safety of physicians, need to be addressed for physicians to be able to provide evidence-based care and to increase the trust of women and communities in the public health system of the country. Policies should be made to provide both quality pre-service and in-service clinical and communication training to improve the skills of physicians and to develop greater confidence in their decision-making. Local opinion leaders such as the Obstetrics and Gynaecologic Society of Bangladesh need to play a proactive role in the introduction of standardized guidelines for CS decision-making. Audits and feedback should be routinely provided across facilities. Physicians who exhibit a high degree of compliance to standard practice and keep the institutional CS rate at an optimum level should be encouraged as role models and well-rewarded.

## Supplementary Information

**Additional file 1: **Good Reporting of A Mixed Methods Study (GRAMMS) checklist.

**Additional file 2:** Link between the current research study and the larger research study in Bangladesh.

**Additional file 3: **Interview Guides.

## Data Availability

The datasets used and/or analyzed during the current study are not available publicly as the data is part of a larger study (where data analysis is still ongoing) but available from corresponding author on reasonable request.

## Abbreviations

C- Section: Cesarean section
NVD: Normal vaginal delivery
WHO: World Health Organization
USG: Ultrasonogram
