# Supplementary material for: Physician–patient communication in decision-making about Caesarean sections in eight district hospitals in Bangladesh: a mixed-method study
Source: Reprod Health. 2021 Feb 9;18:34. doi: 10.1186/s12978-021-01098-8 (PMC7871368; doi:10.1186/s12978-021-01098-8)
Supplement: Supplementary file 1 — Additional file 1: Good Reporting of A Mixed Methods Study (GRAMMS) checklist. [file 12978_2021_1098_MOESM1_ESM.docx]

**GRAMMS checklist**

Describe the justification for using a mixed methods approach to the research question

***Page 3***

Describe the design in terms of the purpose, priority and sequence of methods

***Page 3***

Describe each method in terms of sampling, data collection and analysis

***Pages 4-6***

Describe where integration has occurred, how it has occurred and who has participated in it

***Page 6***

Describe any limitation of one method associated with the present of the other method

***Page 3***

Describe any insights gained from mixing or integrating methods

***Pages 18-19***
