# Supplementary material for: Physician–patient communication in decision-making about Caesarean sections in eight district hospitals in Bangladesh: a mixed-method study
Source: Reprod Health. 2021 Feb 9;18:34. doi: 10.1186/s12978-021-01098-8 (PMC7871368; doi:10.1186/s12978-021-01098-8)
Supplement: Supplementary file 2 — Additional file 2: Link between the current research study and the larger research study in Bangladesh. [file 12978_2021_1098_MOESM2_ESM.docx]

***Additional file 2: Annex 1***

## The link between the current research study and the larger research study in Bangladesh

This PD research study nested within a larger study looking at multiple factors influencing decision-making in C-sections in Bangladesh. The larger study is being conducted on behalf of the Government of Bangladesh and is deemed to be action research through a nexus of researchers and practitioners to understand the reasons behind the rising C-section rates in Bangladesh and to take action to address it.

The design of the larger action research study has been finalised by the Ministry of Health and Family Welfare, Bangladesh, in consultation with select agencies in the country which provide technical assistance to it. The United Nations Population Fund, Bangladesh (UNFPA), is funding the study at the request of the Government of Bangladesh. The PD student works at UNFPA and is part of the advisory team in the design of the larger study. Through a desk review process on research capabilities, the Maternal and Child Health division of the International Centre for Diarrheal Diseases Research, Bangladesh, was given the responsibility of carrying out the overall action research on behalf of the government.

The larger study is not backed by detailed literature review but is based on local contextual factors as determined by perceptions and experience of multiple stakeholders in the country. The larger study casts a wider net in identifying modifiable factors in helping reduce C-section rate in Bangladesh. The PD study shares part of the study settings as the larger study. The PD restricts itself to 8 of the 16 district hospitals of the larger study and does not delve into the sub-district hospital level. The data collected for the PD study also forms part of the pool of data for the larger study. The larger study includes additional participants both in the form of numbers and type (e.g. The larger study covers 592 observations while the PD study covers 296 only; the larger study interviews midwives, nurses and facility managers in addition to the physicians while the PD study involves the physicians only).

A comparison table between the larger study and the PD research study are given below:

|  | Larger study | PD research study |
| --- | --- | --- |
| Study setting | 16 district hospitals  48 sub-district hospitals | 8 out of the 16 district hospitals to be covered in the larger study  None |
| Quantitative methods – sample size | | |
| Method | Larger study | PD research study |
| Observation | 592 | 296 (completed 306) out of the 592 |
| Physician interview | 160 |  |
| Women who delivered | 592 |  |
| Qualitative methods – sample size | | |
| Physician interview | 64 | 16 out of the 64 physicians in total as part of the larger study |
| Women who underwent Emergency C-section | 32 | 16 out of the 32 women to be interviewed in total as part of the larger study |
| Women who underwent Elective C-section | 32 | 16 out of the 32 women to be interviewed in total as part of the larger study |
| Facility Managers | 64 |  |
| Midwives and Nurse Midwives | 16 |  |

The larger quantitative study involved 17 field researchers, and the qualitative study team had six field researchers. 9 out of the 17 quantitative field researchers and all six qualitative field researchers supported data collection for the PD research study component of the larger study. The same researchers would also continue collecting data for the larger study. The eight district hospitals which were part of the PD research study were covered first, and all components of the larger study were completed for each of the 8 facilities in one visit. The larger study continues into the other eight district hospitals and 48 sub-district hospitals and is likely to be completed by mid-2019.

***Additional file 2: Annex 2***

***General Service availability and inpatient services***

| Characteristics | Noakhali | Jessore | Jamalpur | Bogra | M. Bazar | Patuakhali | Panchagarh | Rajbari | Total (%) |
| --- | --- | --- | --- | --- | --- | --- | --- | --- | --- |
| Child vaccination services | 1 | 1 | 0 | 1 | 1 | 1 | 1 | 0 | 6 (75) |
| Growth monitoring services | 1 | 1 | 1 | 0 | 1 | 1 | 1 | 1 | 7 (87.5) |
| Curative care services for children under-age 5 | 1 | 1 | 1 | 1 | 1 | 0 | 1 | 1 | 7 (87.5) |
| Any family planning service | 0 | 1 | 1 | 1 | 1 | 1 | 0 | 1 | 6 (75) |
| Antenatal care (ANC) services | 1 | 1 | 1 | 1 | 1 | 1 | 1 | 1 | 8 (100) |
| Normal delivery | 1 | 1 | 1 | 1 | 1 | 1 | 1 | 1 | 8 (100) |
| Cesarean delivery (Cesarean section) | 1 | 1 | 1 | 1 | 1 | 1 | 1 | 1 | 8 (100) |
| IMCI service | 0 | 1 | 1 | 1 | 1 | 1 | 1 | 1 | 7 (87.5) |
| Kangaroo mother care service | 1 | 1 | 0 | 0 | 1 | 1 | 1 | 1 | 6 (75) |
| Laboratory diagnostic services | 1 | 1 | 1 | 1 | 1 | 1 | 1 | 1 | 8 (100) |
| Blood grouping and typing services | 1 | 1 |  | 1 | 1 | 1 | 1 | 1 | 8 (100) |
| Blood transfusion services | 1 | 1 | 1 | 1 | 1 | 1 | 1 | 1 | 8 (100) |
| Postnatal care (PNC) services | 1 | 1 | 1 | 1 | 1 | 1 | 1 | 1 | 8 (100) |
| Postpartum family planning (PPFP) services | 0 | 1 | 1 | 0 | 1 | 1 | 1 | 0 | 5(62.7) |
| Adolescent health services | 0 | 1 | 1 | 0 | 1 | 1 | 0 | 0 | 4(50) |

0 – Not available; 1 – available

**Inpatient services**

| Characteristics | Noakhali | Jessore | Jamalpur | Bogra | M. Bazar | Patuakhali | Panchagarh | Rajbari | Total (%) |
| --- | --- | --- | --- | --- | --- | --- | --- | --- | --- |
| Provide in-patient care | 1 | 1 | 1 | 1 | 1 | 1 | 1 | 1 | 8 (100) |
| Number of beds excluding maternity/delivery bed | 250 | 235 | 226 | 230 | 230 | 215 | 90 | 84 |  |
| Number of beds according to GOB circular/license | 250 | 250 | 250 | 250 | 250 | 250 | 100 | 100 |  |
| Designated beds for maternity service | 0 | 15 | 24 | 20 | 20 | 35 | 10 | 16 |  |
| Designated bed for pediatrics | 32 | 24 | 24 | 20 | 52 | 58 | 14 | 12 |  |
| Designated beds for newborns | 0 | 4 | 0 | 20 | 15 | 32 | 0 | 0 |  |

***24-hour staff coverage***

| Characteristics | Noakhali | Jessore | Jamalpur | Bogra | M. Bazar | Patuakhali | Panchagarh | Rajbari | Total (%) |
| --- | --- | --- | --- | --- | --- | --- | --- | --- | --- |
| 24 hours health care present at facility | 1 | 1 | 1 | 1 | 1 | 1 | 1 | 1 | 8 (100) |
| 24 hours duty schedule present | 1 | 1 | 1 | 1 | 1 | 1 | 1 | 1 | 8 (100) |

**User fee**

| Characteristics | Noakhali | Jessore | Jamalpur | Bogra | M. Bazar | Patuakhali | Panchagarh | Rajbari | Total (%) |
| --- | --- | --- | --- | --- | --- | --- | --- | --- | --- |
| Have to pay for Normal Delivery | 0 | 0 | 0 | 0 | 0 | 0 | 0 | 0 | 0 |
| Have to pay for CS | 0 | 0 | 0 | 0 | 0 | 0 | 0 | 0 | 0 |
| Official fees are posted or displayed | 1 | 1 | 1 | 1 | 1 | 1 | 1 | 1 | 8 (100) |

**Transport for emergency**

| Characteristics | Noakhali | Jessore | Jamalpur | Bogra | M. Bazar | Patuakhali | Panchagarh | Rajbari | Total (%) |
| --- | --- | --- | --- | --- | --- | --- | --- | --- | --- |
| Functional ambulance | 1 | 1 | 1 | 1 | 1 | 1 | 1 | 1 | 8 (100) |
| Driver available | 1 | 1 | 1 | 1 | 1 | 1 | 1 | 1 | 8 (100) |

***Delivery and newborn care***

| Characteristics | Noakhali | Jessore | Jamalpur | Bogra | M. Bazar | Patuakhali | Panchagarh | Rajbari | Total (%) |
| --- | --- | --- | --- | --- | --- | --- | --- | --- | --- |
| Normal delivery available in this facility | 1 | 1 | 1 | 1 | 1 | 1 | 1 | 1 | 8 (100) |
| Dedicated room for normal delivery | 1 | 1 | 1 | 1 | 1 | 1 | 1 | 1 | 8 (100) |
| Skilled person present 24 hours | 1 | 1 | 1 | 1 | 1 | 1 | 1 | 1 | 8 (100) |
| Duty schedule or call list for 24-hr staff | 1 | 1 | 1 | 1 | 1 | 1 | 1 | 1 | 8 (100) |
| Duty schedule or call list for 24-HR staff observed | 1 | 1 | 1 | 1 | 1 | 1 | 1 | 1 | 8 (100) |

***Delivery and new born care***

| Characteristics | Noakhali | Jessore | Jamalpur | Bogra | M. Bazar | Patuakhali | Panchagarh | Rajbari | Total (%) |
| --- | --- | --- | --- | --- | --- | --- | --- | --- | --- |
| Normal delivery available in this facility | 1 | 1 | 1 | 1 | 1 | 1 | 1 | 1 | 8 (100) |
| Dedicated room for normal delivery | 1 | 1 | 1 | 1 | 1 | 1 | 1 | 1 | 8 (100) |
| Skilled person present 24 hours | 1 | 1 | 1 | 1 | 1 | 1 | 1 | 1 | 8 (100) |
| Duty schedule or call list for 24-hr staff | 1 | 1 | 1 | 1 | 1 | 1 | 1 | 1 | 8 (100) |
| Duty schedule or call list for 24-HR staff observed | 1 | 1 | 1 | 1 | 1 | 1 | 1 | 1 | 8 (100) |

***Signal function***

| Characteristics | Noakhali | Jessore | Jamalpur | Bogra | M. Bazar | Patuakhali | Panchagarh | Rajbari | Total (%) |
| --- | --- | --- | --- | --- | --- | --- | --- | --- | --- |
| Parenteral administration of antibiotics (IV or IM) | 1 | 1 | 1 | 1 | 1 | 1 | 1 | 1 | 8 (100) |
| Parenteral administration of oxytocic (IV or IM) | 1 | 1 | 1 | 1 | 1 | 1 | 1 | 1 | 8 (100) |
| Parenteral administration of anticonvulsant for hypertensive disorders of pregnancy/ prevention of eclampsia (IV or IM) | 1 | 1 | 1 | 1 | 1 | 1 | 1 | 1 | 8 (100) |
| Assisted vaginal delivery | 0 | 1 | 0 | 0 | 1 | 0 | 0 | 1 | 3(37.5) |
| Manual removal of placenta | 1 | 1 | 1 | 1 | 1 | 1 | 1 | 1 | 8 (100) |
| Removal of retained products of conception | 1 | 1 | 1 | 1 | 1 | 1 | 1 | 1 | 8 (100) |
| Neonatal resuscitation | 1 | 1 | 1 | 1 | 1 | 1 | 1 | 1 | 8 (100) |
| Corticosteroids for pre-term labour | 1 | 1 | 1 | 1 | 1 | 1 | 1 | 1 | 8 (100) |

***Labor Room***

| Characteristics | Noakhali | Jessore | Jamalpur | Bogra | M. Bazar | Patuakhali | Panchagarh | Rajbari | Total (%) |
| --- | --- | --- | --- | --- | --- | --- | --- | --- | --- |
| Number of beds | 3 | 2 | 3 | 2 | 2 | 5 | 2 | 2 |  |
| Availability of awareness materials (posters) on WASH | 1 | 1 | 0 | 0 | 1 | 0 | 1 | 0 | 4(50) |
| Maintenance of treatment sheet | 1 | 1 | 1 | 1 | 1 | 1 | 1 | 1 | 8 (100) |
| Maintenance of bed head ticket | 1 | 1 | 1 | 1 | 0 | 0 | 1 | 0 | 5(62.7) |
| Maintenance of temperature chart | 1 | 1 | 1 | 0 | 1 | 1 | 1 | 1 | 7(87.5) |
| Availability of screen in the windows/ doors | 1 | 1 | 0 | 1 | 0 | 1 | 1 | 1 | 6 (75) |
| Availability of running water supply/ functional tape | 1 | 1 | 1 | 1 | 1 | 1 | 1 | 1 | 8 (100) |
| Hand washing facility availability | 1 | 1 | 1 | 1 | 1 | 1 | 1 | 1 | 8 (100) |

***Caesarean Delivery***

| Characteristics | Noakhali | Jessore | Jamalpur | Bogra | M. Bazar | Patuakhali | Panchagarh | Rajbari | Total (%) |
| --- | --- | --- | --- | --- | --- | --- | --- | --- | --- |
| Availability of HCP 24 hours who can perform CS | 1 | 1 | 1 | 1 | 1 | 1 | 1 | 1 | 8 (100) |
| Facility has an anesthetist present in the facility or on call 24 hours a day | 1 | 1 | 1 | 0 | 1 | 1 | 1 | 1 | 7(87.5) |
| Anesthesia machine | 1 | 0 | 0 | 0 | 0 | 0 | 0 | 0 | 1(12.5) |
| Tubing’s and connectors (to connect endotracheal tube) | 0 | 0 | 0 | 1 | 0 | 0 | 0 | 0 | 1(12.5) |
| Oropharyngeal airway (adult) | 0 | 0 | 0 | 0 | 0 | 1 | 0 | 0 | 1(12.5) |
| Oropharyngeal airway (pediatric) | 1 | 0 | 1 | 1 | 1 | 1 | 1 | 1 | 7(87.5) |
| Magill’s forceps - adult | 1 | 1 | 1 | 1 | 1 | 1 | 1 | 1 | 8 (100) |
| Magill’s forceps - pediatric | 1 | 1 | 1 | 0 | 1 | 1 | 1 | 1 | 7(87.5) |
| Endotracheal tube cuffed sizes 3.0 - 5.0 | 1 | 1 | 1 | 0 | 0 | 1 | 1 | 1 | 6 (75) |
| Endotracheal tube cuffed sizes 5.5 - 9.0 | 1 | 1 | 1 | 0 | 0 | 1 | 1 | 1 | 6 (75) |
| Intubating stylet | 1 | 0 | 1 | 1 | 1 | 1 | 1 | 1 | 7(87.5) |
| Spinal needle | 1 | 0 | 0 | 1 | 0 | 1 | 1 | 1 | 5(62.7) |
| OT table | 1 | 1 | 1 | 1 | 1 | 1 | 1 | 1 | 8 (100) |
| OT light | 1 | 1 | 1 | 1 | 1 | 1 | 1 | 1 | 8 (100) |
| IV stand | 1 | 1 | 1 | 1 | 1 | 1 | 1 | 1 | 8 (100) |
| Emergency power supply | 1 | 1 | 1 | 1 | 1 | 1 | 1 | 1 | 8 (100) |
| Instrument set for caesarean-delivery | 1 | 1 | 1 | 1 | 1 | 1 | 1 | 1 | 8 (100) |
| Air conditioner | 1 | 1 | 1 | 1 | 1 | 1 | 1 | 1 | 8 (100) |
| Oxygen cylinder with flow meter | 1 | 1 | 1 | 1 | 1 | 1 | 1 | 1 | 8 (100) |
| Oxygen cylinder without flow meter | 1 | 0 | 0 | 0 | 1 | 1 | 1 | 0 | 4(50) |
| Sterile gloves | 1 | 1 | 1 | 1 | 1 | 1 | 1 | 1 | 8 (100) |
| Disinfectant | 1 | 1 | 0 | 1 | 0 | 1 | 1 | 1 | 6 (75) |

***Blood transfusion services***

| Characteristics | Noakhali | Jessore | Jamalpur | Bogra | M. Bazar | Patuakhali | Panchagarh | Rajbari | Total (%) |
| --- | --- | --- | --- | --- | --- | --- | --- | --- | --- |
| Blood transfusion available in this facility | 1 | 1 | 1 | 1 | 1 | 1 | 1 | 1 | 8 (100) |
| screening done before transfusion |  |  |  |  |  |  |  |  |  |
| HIV | 1 | 1 | 1 | 1 | 1 | 1 | 1 | 1 | 8 (100) |
| Syphilis/ VDRL | 1 | 1 | 1 | 1 | 1 | 1 | 1 | 1 | 8 (100) |
| Hepatitis B | 1 | 1 | 1 | 1 | 1 | 1 | 1 | 1 | 8 (100) |
| Hepatitis C | 1 | 1 | 1 | 1 | 1 | 1 | 1 | 1 | 8 (100) |
| Malaria | 1 | 1 | 1 | 1 | 1 | 1 | 1 | 1 | 8 (100) |

***Staffing***

| Designation | Noakhali | | Jessore | | Jamalpur | | Bogra | | M.Bazar | | Patuakhali | | Panchagarh | | Rajbari | |
| --- | --- | --- | --- | --- | --- | --- | --- | --- | --- | --- | --- | --- | --- | --- | --- | --- |
|  | Sanc | posted | Sanc | posted | Sanc | posted | Sanc | posted | Sanc | posted | Sanc | posted | Sanc | posted | Sanc | posted |
| Sr. Consultant (Obs. & Gynecology) | 0 | 0 | 0 | 0 | 1 | 0 | 1 | 0 | 1 | 1 | 1 | 1 | 1 | 0 | 1 | 0 |
| Jr. Consultant (Obs. & Gynecology) | 1 | 1 | 1 | 1 | 1 | 1 | 2 | 2 | 1 | 1 | 1 | 1 | 1 | 1 | 1 | 1 |
| Sr. Consultant (Paedi) | 1 | 1 | 1 | 0 | 1 | 0 | 1 | 0 | 1 | 1 | 1 | 1 | 1 | 0 | 1 | 1 |
| Jr. Consultant (Paedi) | 0 | 0 | 1 | 0 | 1 | 1 | 1 | 1 | 1 | 0 | 1 | 0 | 1 | 1 | 1 | 0 |
| Sr. Consultant (Anesthetist) | 1 | 0 | 0 | 0 | 1 | 0 | 0 | 0 | 1 | 0 | 1 | 1 | 0 | 0 | 1 | 0 |
| Jr. Consultant (Anesthetist) | 1 | 1 | 1 | 0 | 1 | 1 | 1 | 1 | 1 | 1 | 0 | 1 | 1 | 1 | 1 | 1 |
| Consultant (Radiology and imaging) | 1 | 1 | 1 | 1 | 1 | 0 | 1 | 1 | 1 | 1 | 1 | 0 | 1 | 0 | 1 | 0 |
| Consultant (Pathologist) | 1 | 0 | 1 | 1 | 1 | 0 | 1 | 0 | 1 | 0 | 1 | 0 | 0 | 0 | 1 | 0 |
| Medical officer (physician) | 11 | 9 | 6 | 5 | 1 | 0 | 14 | 14 | 6 | 5 | 10 | 1 | 4 | 3 | 4 | 4 |
| Radiologist | 1 | 0 | 1 | 1 | 1 | 0 | 1 | 1 | 1 | 1 | 1 | 0 | 1 | 0 | 1 | 1 |
| Pathologist | 1 | 1 | 1 | 1 | 1 | 0 | 2 | 1 | 1 | 0 | 1 | 0 | 1 | 0 | 1 | 0 |
| Senior clinical pathologist | 0 | 0 | 0 | 0 | 0 | 0 | 0 | 0 | 0 | 0 | 1 | 0 | 0 | 0 |  | 0 |
| Anesthetist | 1 | 1 | 1 | 1 | 1 | 1 | 2 | 1 | 3 | 2 | 3 | 0 | 1 | 0 | 0 | 0 |
| Emergency medical officer (EMOs) | 6 | 4 | 4 | 3 | 4 | 3 | 1 | 7 | 3 | 3 | 4 | 0 | 3 | 1 | 0 | 0 |
| Indoor medical officer-Gynae & obs. | 0 | 0 | 2 | 2 | 1 | 1 | 1 | 1 | 0 | 0 | 0 | 0 | 1 | 1 | 0 | 0 |
| Assist register –Gynae & obs. | 2 | 2 | 2 | 2 | 2 | 0 | 4 | 1 | 2 | 2 | 2 | 1 | 0 | 0 | 0 | 0 |
| SACMO (sub- assistant community medical officer | 0 | 0 | 0 | 0 | 0 | 0 | 0 |  | 0 | 0 | 0 | 0 | 0 | 0 | 0 | 0 |
| Family welfare visitor (FWV) | 0 | 0 | 0 | 0 | 0 | 0 | 0 | 0 | 0 | 0 | 0 | 0 | 0 | 0 | 0 | 0 |
| Nursing supervisor | 5 | 3 | 4 | 1 | 4 | 2 | 10 | 7 | 4 | 3 | 4 | 3 | 2 | 2 | 2 | 1 |
| Senior staff nurse | 153 | 100 | 199 | 199 | 157 | 119 | 114 | 112 | 111 | 79 | 98 | 80 | 54 | 47 | 56 | 56 |
| Nurse midwife | 0 | 0 | 0 | 0 | 0 | 0 | 0 |  | 0 | 0 | 0 | 0 | 0 | 0 | 0 | 0 |
| Staff nurse | 24 | 16 | 35 | 35 | 14 | 11 | 61 | 56 | 12 | 6 | 16 | 14 | 11 | 4 | 11 | 11 |
| Assistant nursing attendant | 7 | 5 | 6 | 2 | 6 | 4 | 6 | 4 | 1 | 1 | 6 | 3 | 5 | 0 | 5 | 3 |
| Midwife | 0 | 0 | 0 | 0 | 0 | 0 | 0 | 0 | 0 | 0 | 0 | 0 | 0 | 0 | 0 |  |
| Medical technologist (LAB) | 3 | 2 | 2 | 1 | 2 | 2 | 2 | 2 | 2 | 2 | 2 | 2 | 2 | 1 | 2 | 2 |
| Medical technologist (Blood transfusion) | 1 | 1 | 1 | 1 | 2 | 2 | 2 | 2 | 1 | 1 | 1 | 1 | 0 | 0 | 0 | 0 |
| Medical technologist (radiology) | 1 | 1 | 1 | 1 | 1 | 0 | 3 | 3 | 2 | 1 | 2 | 2 | 2 | 0 | 2 | 2 |

**Total deliveries as reported in the previous 6 months *(March to August 2018)***

| **Month** | **Bogra** | | **Jamalpur** | | **Jessore** | | **M. Bazar** | |
| --- | --- | --- | --- | --- | --- | --- | --- | --- |
|  | CS | NVD | CS | NVD | CS | NVD | CS | NVD |
| March | 104(77.6%) | 30(22.4%) | 87(49.2%) | 90(50.8%) | 124(56.9%) | 94(43.1%) | 39(17.0%) | 191(83.0%) |
| April | 115(74.7%) | 39(25.6%) | 77(50.3%) | 76(49.7%) | 102(42.1%) | 140(57.9%) | 57(25.9%) | 163(74.1%) |
| May | 88(62%) | 54(38%) | 71(44.4%) | 89(55.6% | 125(53.4%) | 109(46.6%) | 42(21.3%) | 155(78.7%) |
| June | 87(65.4%) | 46(34.6%) | 64(46%) | 75(54%) | 98(47.6%) | 108(52.4%) | 34(20.6%) | 131(79.4%) |
| July | 104(80.6%) | 25(19.4%) | 73(40.8%) | 106(59.2%) | 127(50.6%) | 124(49.4%) | 49(29.7%) | 116(70.3%) |
| August | 110(75.9%) | 35(24.1%) | 65(51.6%) | 61(48.4%) | 133(51.0%) | 128(49.0%) | 52(22.9%) | 175(77.1% |
| Total | 608(72.6) | 229(27.4) | 437(46.8) | 497(53.2) | 709(50.2) | 703(49.8) | 307(24.8) | 931(75.2) |

| **Month** | **Noakhali** | | **Panchagarh** | | **Patuakhali** | | **Rajbari** | |
| --- | --- | --- | --- | --- | --- | --- | --- | --- |
|  | CS | NVD | CS | NVD | CS | NVD | CS | NVD |
| March | 169(56.1%) | 132(43.9%) | 49(32.7%) | 101(67.3%) | 86(44.6%) | 107(54.4%) | 47(37.3%) | 79(62.7%) |
| April | 156(55.1%) | 127(44.9%) | 45(42.1%) | 62(57.8%) | 92(53.5%) | 80(46.5%) | 42(39.3%) | 65(60.7%) |
| May | 179(55.8%) | 142(44.2%) | 28(27.2%) | 75(72.8%) | 86(48.6%) | 91(51.4%) | 40(33.3%) | 80(56.7%) |
| June | 131(44.6%) | 163(55.4%) | 27(26%) | 67(74%) | 73(48.3%) | 78(51.7%) | 36(26.7%) | 99(73.3%) |
| July | 149(47%) | 168(53%) | 30(28.8%) | 74(72.2%) | 87(55.1% | 71(44.9%) | 46(34.1%) | 89(65.9%) |
| August | 156(46.8%) | 177(53.2%) | 24(28.6%) | 60(72.4%) | 85(45.5% | 102(54.5%) | 33(28.2%) | 84(71.8%) |
| Total | 940(50.8) | 909(49.2) | 203(31.6) | 439(68.4) | 509(49.0) | 529(51.0) | 244(33.0) | 496(67.0) |
