# Supplementary material for: Physician–patient communication in decision-making about Caesarean sections in eight district hospitals in Bangladesh: a mixed-method study
Source: Reprod Health. 2021 Feb 9;18:34. doi: 10.1186/s12978-021-01098-8 (PMC7871368; doi:10.1186/s12978-021-01098-8)
Supplement: Supplementary file 3 — Additional file 3: Interview Guides. [file 12978_2021_1098_MOESM3_ESM.docx]

***Additional file 3: Annex 1***.

**Guideline for Consultant/Physicians**

After completing the face sheet:

***Introduction***

1. Can you tell me a bit about your background – where you come from, your education, designation, how long you’ve been in this hospital etc.?
2. Can you give me details of any special trainings you have had in your career?
3. How would you rate your post graduate training in obstetrics?

Probing

- NVD
- C-section
- Assisted delivery
- Use of Partograph

1. Can you tell me about your obstetric history?

Probing

- Focus on modes of delivery and how those decisions were made?

***Exploring C-section decision-making***

1. What do you think are the factors responsible for increasing trend of C-section?

Probing

- - **Provider perspective**
    - Financial benefit
    - Workload
    - Private practice
    - Fear of blame for any adverse effect due to NVD/assisted delivery
  - **Health systems factors**
    - Staffing
    - Training
    - Infrastructure
    - Referral
  - **Patient factors**
    - Pain
    - Convenience

1. How do you generally decide mode of delivery (NVD/ Assisted VD/ C-section)? What factors do you consider?
2. What are the protocols do you use while making decisions?
3. How do you communicate with patients while informing them about the decision regarding mode of delivery?

Probing

- What do you tell them?
- What do patients or relatives ask?
- What do you think are the facilitators and barriers of communication in such situations?

1. Have you received any specialized training for communication and particularly communication on mode of delivery?
2. What are challenges you face during decision-making? (Facility readiness, patient’s personal preferences etc.)

***Conclusion:***

1. Is there anything more you would like to add?

**Guideline for recently delivered (by C-section) mother**

***Introduction***

1) Can you tell me a bit about your background – where you come from, your education etc.?

2) Can you tell me about your obstetric history?

3) Can you walk me through your current pregnancy? All service providers you met, ANC, any trial at home etc.

Probing:

- Where did you get ANC and other pregnancy related care?
- Why did you decide to come to this hospital? Can you explain what were the circumstances preceding arrival in this hospital?

4) What were your expectations for this pregnancy (in terms of mode of delivery); did you have prior knowledge on the pros and cons of NVD and C-sections

5) Can you explain what happened after you reached this hospital?

6) What has your experience been in this hospital?

- Privacy, respect and information provided by service providers
- Money spent

***Exploring C-section decision-making***

7) Who made the decision regarding C- Section?

Probing:

- You or others (doctor, midwives, family members etc.)?
- Can you explain the time when the decision was made? What did the physician say? What did you say? Did anyone else say anything (nurse, midwife, your family members, other hospital staff, others)?

8) (If you took the decision) what are the factors which influenced your decision regarding C-section?

Probing:

- Fear of pain
- Fear of episiotomy
- Safety issues
- Negative birth experience
- Specific belief
- Convenience
- Others

9) (If another person took the decision) why did they decide to go for a C-section?

Probing:

- Why did they decide so?
- Do you know the indication for which C-section was performed?

10) What was the process of decision-making?

Probing:

- Details provided and preference taken
- Involvement of family members

11) Can you explain the consenting process?

Probing:

- Did you sign the consent form or someone else?
- Did you fully understand what was in the consent form?
- Did anyone explain the pros and cons of the options you had?
- Did you have any questions for them? Were they answered?

***Conclusion:***

1. Is there anything more you would like to add?

***Additional file 3: Annex 2a***: **Physician interview codes and themes**

| **Codes** | **Category** | **Final theme** | **Context** |
| --- | --- | --- | --- |
| Workload; Night hours; Private practice; Wide job description; Role as a mother; Normal delivery takes time; Personal security and lack of transport; Role as information provider on complications; Morning only surgeries | Personal and professional workload balance | Work-life balance | From within |
| Type of delivery is a feeling; Normal can be risky to the baby; I will decide; Save mother’s lives; Personal experience; Normal delivery is best; Not a topic of personal choice; Limited trainings; Uncertainty on indications for C-section; Europe model not possible Rates unaware; Couldn’t follow protocol; Patients rely on us and agree with my decision | Physician experience and perceptions | Personal preferences |  |
| Middlemen influence the decision; Role of nurses and other co-workers; media; Politicians; Risk of harassment; Community acceptance of C-Section as new normal; Midwives help; Trial at home – TBAs (indiscriminate use of oxytocin); Grandmothers and mothers pressure; Privacy; People losing tolerance power; Referral needs money Too many attendants | External influence | External influence | From without |
| Risk aversion linked to country culture; Outside countries – can do trial; Uterine rupture as a serious risk; Self-referral to other facilities; Patients worry when physicians not around | Fear and Risk Aversion | Risk Aversion | System and skills |
| Communication –very sensitive situation; Difficult to motivate; Consent needed to prevent accusation later; Nothing without consent; Mother emotional in emergencies; She has the right to know the reason; Telling; counselling and convincing; Illiteracy | Communication as a way of sharing information | Communication skills |  |
| Staff shortage; Human resources lack; oxytocin at home; No ICU; No specialists; Tools for decision-making; Anesthetists; Autoclaves; No instruments for assisted delivery; No epidural; Electricity/Generator; Everything is linked; Blood; No continuous monitoring | Human resource challenges | Health system |  |

***Additional file 3: Annex 2b***: **Women – Emergency C-section interview codes and themes**

| **Codes** | **Category** | **Final theme** | **Context** |
| --- | --- | --- | --- |
| Attempts at home; Traditional birth attendants; past negative experience | Local pressure | Yielding to local pressure | Guilt |
| The behaviour of health care providers, rudeness, aggression, yelling, not listening, had to obey | Health workers attitude | Lack of respect | Powerlessness |
| Myths and misconceptions – videos, big baby, high BP, water break, assumptions on physician availability, multiple service provider contact physicians taking religious angle; more effort on dissuading preferred mode of delivery. | Confidence in indications | Speaking the same language on indications | Knowledge |
| Fear and fright, mother’s death, baby’s death | Negative information exchange | Negative language | Language |
| Overhearing; no care talk but direct cure talk; | Interpretation skills of the woman | Technical language |  |
| Cost driving request; have spent a lot and nothing more left; home too to come back; no option | Do what you can | Prayers take over | Fatalism |
| Too much uncertainty and cannot handle the pressure | Emotional drain | Decision under pressure/ Quick end |  |

***Additional file 3: Annex 2c:*** **Women – Elective C-section interview codes and themes**

| **Codes** | **Category** | **Final theme** | **Context** |
| --- | --- | --- | --- |
| Allah knows better; Traditional healer (Kabiraz); Alga Talga (Devil air); Blessing from elderly people; Faith on almighty; Myths of evil spirits | Faith and resigned to a destiny | Faith | Safety of C-sections |
| Ultrasonogram (USG) at Private clinic and its centrality in fixing indications:  Baby’s position wasn’t good; Baby was weak; Baby movement was less; Rupture of membranes; Post-date | USG and its universality  for determining indications | USG and its universality |  |
| Learnt from other people; Younger sister; aunty on safety; Only heavy work after C-section not possible | Sources of information | Confidence in safety |  |
| Whatever they suggest; We have nothing to say except arranging blood – clue for C-section; Consent to protect themselves from a claim for death; No more information | One-way (limited) communication | Physicians know best | Physicians in control |
| Illiteracy; Poverty; Don’t know about consent; Don’t care; Don’t know why we signed | Consenting without understanding | Consent, a formality |  |
| Sterilization; Man is working; Distance – no point returning | An added benefit of combining sterilization | Collateral benefits | Value for money |
| Anything for my baby; God’s gift; Don’t want to take the risk; Previous negative experience | Complications don’t matter | Baby is the future | Sacrificial attitude |
| Hospital environment; fear of pain; seeing others cry | Lack of privacy fuelling fear | Privacy over pain | Fear of pain – not a major concern |
